# Effectiveness of SGLT2 inhibitors compared to sulfonylureas for long-term glycemic control in type 2 diabetes: A meta-analysis

**DOI:** 10.17305/bb.2025.12658

**Published:** 2025-07-03

**Authors:** Zhouhong Zhan, Jialiang Wang, Nannan Shen, Xinwen Liu, Lihong Wang

**Affiliations:** 1 Clinical Pharmacy Department, The Affiliated Hospital of Shaoxing University, Shaoxing, Zhejiang Province, China

**Keywords:** Sodium-glucose co-transporter 2, SGLT2 inhibitors, sulfonylureas, SUs, glycemic durability, HbA1c, meta-analysis, type 2 diabetes mellitus, T2DM

## Abstract

Sulfonylureas (SUs) are common glucose-lowering agents used for managing type 2 diabetes mellitus (T2DM). However, their long-term effectiveness is often limited due to declining β-cell function. Sodium-glucose co-transporter 2 (SGLT2) inhibitors act independently of insulin, potentially providing more sustained glycemic control. Nonetheless, comparative data regarding the long-term glycemic durability of these two drug classes are limited. We performed a meta-analysis of head-to-head randomized controlled trials (RCTs) comparing the efficacy of SGLT2 inhibitors versus SUs in patients with T2DM already receiving metformin therapy. Eligible studies reported HbA1c values at intermediate (24–28 weeks or 48–52 weeks) and final (96–104 weeks or 208 weeks) time points, with a minimum follow-up duration of 96 weeks. Pooled mean differences (MD) and their 95% confidence intervals (CIs) were calculated using random-effects models. Seven comparisons from five RCTs were included in our analysis. Compared with SUs, SGLT2 inhibitors were associated with significantly smaller increases in HbA1c over time. From 24–28 weeks to 96–104 weeks, the pooled MD was −0.28% (95% CI: −0.35 to −0.20; *P* < 0.001; *I*^2^ ═ 0%). From 48–52 weeks to 96–104 weeks, the MD was −0.11% (95% CI: −0.19 to −0.04; *P* ═ 0.004; *I^2^* ═ 0%). In longer-term analyses, SGLT2 inhibitors demonstrated sustained benefits from 52 weeks to 208 weeks (MD: −0.22%; 95% CI: −0.34 to −0.10; *P* < 0.001) and from 104 weeks to 208 weeks (MD: −0.12%; 95% CI: −0.25 to −0.01; *P* ═ 0.04). Overall, SGLT2 inhibitors provide superior glycemic durability compared to SUs in patients with T2DM, supporting their preferential use as a second-line therapy after metformin.

## Introduction

Type 2 diabetes mellitus (T2DM) is a chronic metabolic disorder characterized by insulin resistance and progressive pancreatic β-cell dysfunction [1, 2]. It currently affects over 500 million individuals worldwide—a number projected to rise substantially in the coming decades [3, 4]. The global increase in obesity, sedentary lifestyles, and aging populations contributes significantly to the growing burden of T2DM, which is associated with serious microvascular and macrovascular complications, including retinopathy, nephropathy, cardiovascular disease, and stroke [5]. Achieving and maintaining optimal glycemic control is essential for reducing the risk of these complications and improving long-term outcomes [6].

Sulfonylureas (SUs), one of the oldest classes of oral antidiabetic drugs, are widely used as second-line therapy after metformin due to their rapid and potent glucose-lowering effect [7]. They stimulate insulin secretion from pancreatic β-cells independently of glucose levels, resulting in effective short-term reductions in glycated hemoglobin (HbA1c) [8]. However, their clinical utility is limited by common adverse effects such as hypoglycemia and weight gain, as well as concerns about diminishing efficacy over time [9]. This phenomenon of reduced long-term effectiveness, often referred to as “secondary failure,” highlights the importance of assessing not only a drug’s initial glucose-lowering capacity but also the durability of its effect over time [10, 11]. Glycemic durability refers to a therapy’s ability to maintain glycemic control over the long term [12]. Durable treatments are especially valuable in the context of T2DM’s progressive nature, marked by ongoing β-cell deterioration [13]. In recent years, sodium-glucose co-transporter 2 (SGLT2) inhibitors have emerged as a novel class of antihyperglycemic agents [14]. These drugs lower blood glucose levels by promoting urinary glucose excretion through inhibition of glucose reabsorption in the renal proximal tubules [14, 15]. Unlike SUs, the glucose-lowering action of SGLT2 inhibitors is insulin-independent and therefore does not place additional stress on β-cells [15]. Clinical trials have demonstrated that SGLT2 inhibitors not only improve glycemic control but also promote weight loss and reduce cardiovascular and renal risks, leading to their growing inclusion in treatment guidelines [16]. The superior glycemic durability of SGLT2 inhibitors compared to traditional agents like SUs is biologically plausible and increasingly supported by evidence. Mechanistically, SGLT2 inhibitors may reduce glucotoxicity, oxidative stress, and β-cell overload—factors known to contribute to the progressive decline in insulin secretion in T2DM [17–19]. By alleviating these stressors, they may help preserve β-cell function and sustain glycemic control over time [19]. Although several randomized controlled trials (RCTs) have directly compared SGLT2 inhibitors with SUs [20–26], findings have been variable, and no prior meta-analysis has comprehensively synthesized long-term data on their relative glycemic durability. Therefore, we conducted a meta-analysis of head-to-head RCTs to compare the long-term glycemic durability of SGLT2 inhibitors vs SUs in patients with T2DM.

## Materials and methods

During the design and implementation of this study, we followed the guidelines set forth by PRISMA (Preferred Reporting Items for Systematic Reviews and Meta-Analyses) [27, 28] and the Cochrane Handbook [29]. The protocol for this meta-analysis has been registered with PROSPERO under the registration number CRD420251024614.

### Study inclusion and exclusion criteria

This meta-analysis included studies that met the inclusion criteria specified in the PICOS principle.

P (Patients): Adult patients with the diagnosis of T2DM.

I (Intervention): SGLT2 inhibitors at any approved dose.

C (Control): SUs at any approved dose.

O (Outcome): Durability of glycemic control was assessed by examining the change in HbA1c from 24–28 weeks (early stabilization) or 48–52 weeks (midpoint) to the final time point at 96–104 weeks. For studies with available long-term data (up to 208 weeks), exploratory analyses were conducted comparing HbA1c changes from intermediate (52 or 104 weeks) to 208 weeks.

S (Study design): RCTs with a parallel design and a minimum follow-up duration of 96 weeks, published as full-text articles in English.

Excluded from the analysis were reviews, editorials, studies not designed as RCTs, studies involving patients with type 1 diabetes, those not including SGLT2 inhibitors as an intervention, those not including SUs as controls, or those not reporting the outcomes of interest. If studies with overlapping patient populations were retrieved, the one with the largest sample size was selected for the meta-analysis.

### Database search

The Medline (PubMed), Embase (Ovid), Cochrane Library (CENTRAL), and Web of Science databases were searched using the following combination of terms: (1) “sodium glucose transporter 2 inhibitor” OR “sodium glucose transporter II inhibitor” OR “SGLT 2 inhibitor” OR “SGLT-2 inhibitor” OR “SGLT2” OR “sodium glucose cotransporter 2 inhibitors” OR “canagliflozin” OR “dapagliflozin” OR “empagliflozin” OR “ertugliflozin” OR “tofogliflozin” OR “bexagliflozin” OR “henagliflozin” OR “ipragliflozin” OR “licogliflozin” OR “luseogliflozin” OR “remogliflozin” OR “sergliflozin” OR “sotagliflozin”; (2) “glimepiride” OR “glipizide” OR “gliclazide” OR “glibenclamide” OR “glyburide” OR “gliguidone” OR “sulfonylureas” OR “sulfonylureas”; and (3) “random” OR “randomly” OR “randomized” OR “randomised”, limited to clinical studies involving human subjects. Only studies that included human participants and were published in English were considered. The full search strategy for each database is outlined in Supplemental File 1. Additionally, references from related reviews and original articles were screened as part of the final database search. The final search was conducted on March 11, 2025.

### Data collection and quality evaluation

The two authors independently conducted database searches, data collection, and quality assessments. In case of disagreements, discussions were held with the corresponding author to reach a consensus. The data collected included various aspects, such as overall study information (e.g., first author, publication year, and clinical trial registration details), study design (e.g., double-blind or single-blind), participant characteristics (e.g., number of T2DM patients, mean age, sex, baseline HbA1c, duration of diabetes, and concurrent medications), as well as individual medications and dosages for the intervention group of SGLT2 inhibitors and the control group of SUs. Additionally, intermediate and final time points for evaluating glycemic durability were noted. The quality of the included RCTs was assessed using the Cochrane Risk of Bias Tool, which evaluates aspects such as random sequence generation, allocation concealment, blinding of participants and outcome assessment, handling of incomplete outcome data, selective reporting, and other potential sources of bias. Two reviewers also evaluated the certainty of evidence using the GRADE (Grading of Recommendations, Assessment, Development, and Evaluation) system, which considers risk of bias, inconsistency, indirectness, imprecision, and publication bias [30]. The certainty of evidence was classified as very low, low, moderate, or high. Any disagreements were resolved through discussions with the corresponding author.

### Statistical analysis

The primary effect measure was the mean difference (MD) in HbA1c change from intermediate to final time points between the SGLT2 inhibitor and SU groups, with corresponding 95% confidence intervals (CIs). If HbA1c data at a specified intermediate time point were unavailable, values from the closest available time point were used for analysis. For studies with multiple SGLT2 inhibitor doses, each dose group was treated as a separate comparison, and the sample size of the shared SU comparator arm was evenly divided according to Cochrane guidelines [29]. Heterogeneity was assessed using the Cochrane *Q* test [29], and the *I^2^* statistic was also calculated. *I^2^* values of < 25%, 25%–75%, and > 75% indicated mild, moderate, and substantial heterogeneity, respectively [31]. A random-effects model was used to pool the results, as this model could account for the potential influence of heterogeneity [29]. Sensitivity analysis was performed by excluding one dataset at a time to assess the robustness of the findings [29]. Publication bias was evaluated through visual inspection of funnel plots and by performing Egger’s regression asymmetry test [32]. A *P* < 0.05 was considered statistically significant. Statistical analyses were conducted using RevMan (Version 5.1; Cochrane, Oxford, UK) and Stata software (Version 17.0; Stata Corporation, College Station, TX, USA).

## Results

### Literature search

**Figure 1.**
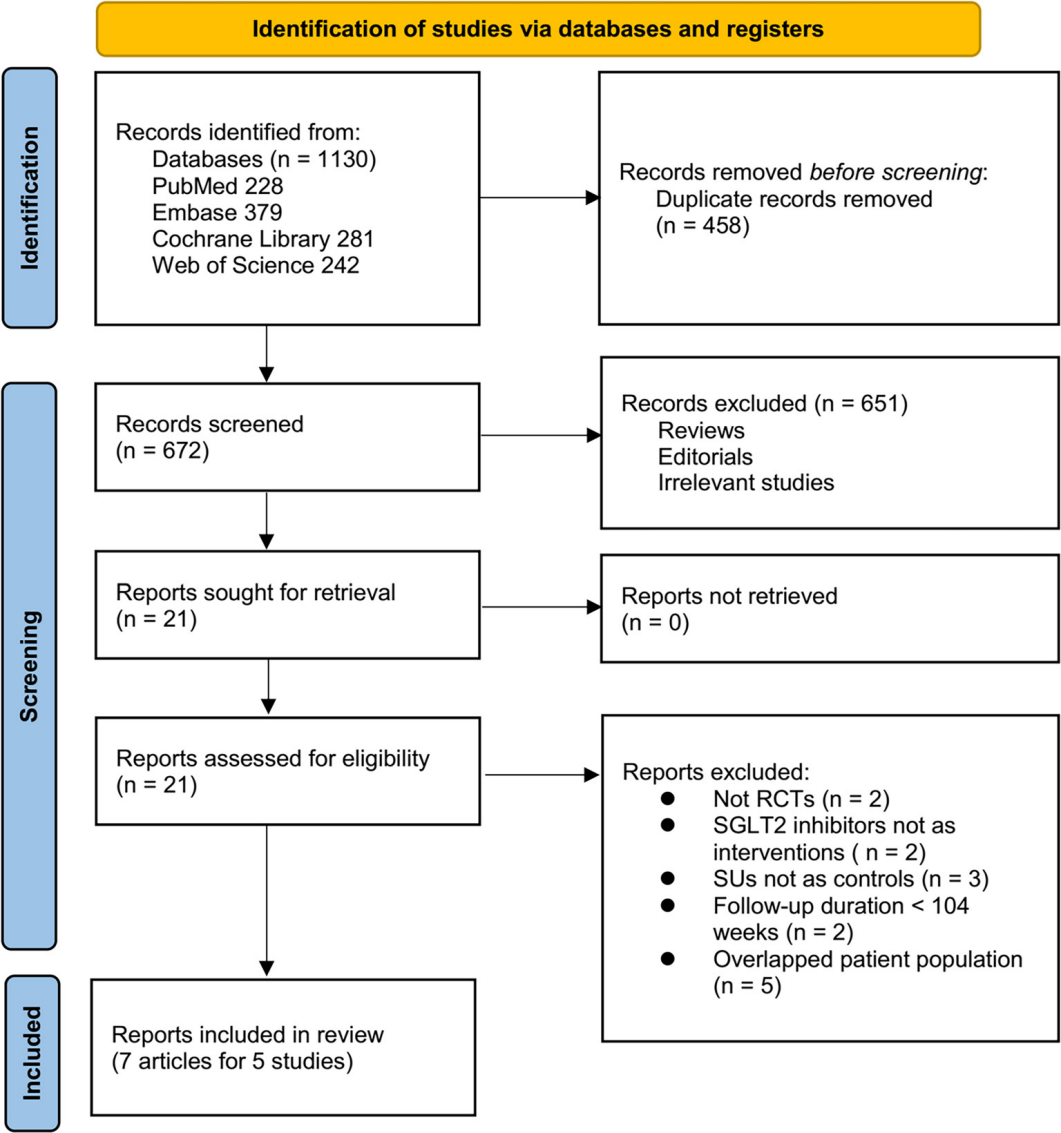
Flowchart for the literature search and study inclusion.

Figure 1 depicts the flowchart outlining the process of database searching and study identification, ultimately leading to the selection of studies for inclusion. Initially, a total of 1,130 articles were retrieved through the database search, which was then reduced to 672 after eliminating 458 duplicate records. Following this, 651 articles were excluded based on an evaluation of their titles and abstracts, primarily due to their lack of relevance to the objective of this meta-analysis. Subsequently, 14 out of the remaining 21 articles were excluded after full-text reviews, for reasons detailed in Figure 1. Ultimately, seven articles from five RCTs were included in the meta-analysis. Of these, five articles reported 96–104-week outcomes for all five RCTs [20, 21, 23, 25, 26], while two additional articles provided 208-week data for two of these trials [22, 24].

### Study characteristics and data quality

An overview of the included studies is provided in Table 1. All studies were multinational, multicenter RCTs involving adult patients with T2DM. Since two studies evaluated different doses of SGLT2 inhibitors [23, 25], each dose group was analyzed separately, resulting in a total of seven datasets in the meta-analysis. In total, 5,550 patients with T2DM were included. The mean age of participants ranged from 55.9–59.6 years, and the proportion of men ranged from 46.5% to 58.2%. The mean baseline HbA1c ranged from 7.7% to 8.0%, and the mean duration of diabetes was between 6.3 and 8.7 years. All participants received concurrent metformin. A total of 3,232 patients were assigned to the treatment group, receiving SGLT2 inhibitors, including dapagliflozin, empagliflozin, canagliflozin, ertugliflozin, and bexagliflozin, while 2,318 patients were allocated to the control group, receiving SUs, including glipizide and glimepiride. The study quality evaluation for the RCTs is detailed in Table 2. All included studies were double-blind RCTs with adequate reporting of random sequence generation and allocation concealment, and were judged to have a low risk of bias across all domains.

**Table 1:** Characteristics of the included RCTs

**Study**	**Registration**	**Design**	**Patient number**	**Mean age (years)**	**Male (%)**	**Baseline HbA1c (%)**	**T2DM duration (years)**	**Concurrent antidiabetic treatment**	**SGLT2 inhibitors and dosages**	**SUs and dosages**	**Intermediate time point (weeks)**	**Final time point (weeks)**
Nauck, 2014	NCT00660907	R, DB	814	58.4	54.2	7.7	6.3	Metformin	Dapagliflozin 5 or 10 mg/d	Glipizide 10–20 mg/d	26, 52	104, 208
Ridderstrale, 2014	NCT01167881	R, DB	1545	55.9	55.2	7.9	6.8	Metformin	Empagliflozin 25 mg/d	Glimepiride 1–4 mg/d	28, 52	104, 208
Leiter, 2015a	NCT00968812	R, DB	724	56.4	55.8	7.8	6.6	Metformin	Canagliflozin 100 mg/d	Glimepiride up to 8 mg/d	26, 52	104
Leiter, 2015b	NCT00968812	R, DB	726	56.2	51.2	7.8	6.7	Metformin	Canagliflozin 300 mg/d	Glimepiride up to 8 mg/d	26, 52	104
Hollander, 2019a	NCT01999218	R, DB	663	58.4	51.1	7.8	7.4	Metformin	Ertugliflozin 5 mg/d	Glimepiride up to 6–8 mg/d	26, 52	104
Hollander, 2019b	NCT01999218	R, DB	652	58.0	46.5	7.8	7.4	Metformin	Ertugliflozin 15 mg/d	Glimepiride up to 6–8 mg/d	26, 52	104
Halvorsen, 2023	NCT03115112	R, DB	426	59.6	58.2	8.0	8.7	Metformin	Bexagliflozin 20 mg/d	Glimepiride up to 6 mg/d	24, 48	96

Abbreviations: RCTs: Randomized controlled trials; HbA1c: Hemoglobin A1c; T2DM: Type 2 diabetes mellitus; SGLT2: Sodium-glucose co-transporter 2; SUs: Sulfonylureas; R: Randomized; DB: Double-blind.

**Table 2:** Study quality evaluation via the Cochrane Risk of Bias Tool

**Study**	**Random sequence generation**	**Allocation concealment**	**Blinding of participants**	**Blinding of outcome assessment**	**Incomplete outcome data addressed**	**Selective reporting**	**Other sources of bias**
Nauck, 2014	Low risk	Low risk	Low risk	Low risk	Low risk	Low risk	Low risk
Ridderstrale, 2014	Low risk	Low risk	Low risk	Low risk	Low risk	Low risk	Low risk
Leiter, 2015a	Low risk	Low risk	Low risk	Low risk	Low risk	Low risk	Low risk
Leiter, 2015b	Low risk	Low risk	Low risk	Low risk	Low risk	Low risk	Low risk
Hollander, 2019a	Low risk	Low risk	Low risk	Low risk	Low risk	Low risk	Low risk
Hollander, 2019b	Low risk	Low risk	Low risk	Low risk	Low risk	Low risk	Low risk
Halvorsen, 2023	Low risk	Low risk	Low risk	Low risk	Low risk	Low risk	Low risk

**Table 3:** Summarized certainty of evidence using the GRADE system

**Outcome**	**Quality assessment**							**Absolute effect** **MD (95% CI)**	**Quality**
	**No. of comparisons**	**Design**	**Risk of bias**	**Inconsistency**	**Indirectness**	**Imprecision**	**Other considerations**		
Difference of HbA1c change from 24∼28 weeks to 96∼104 weeks between SLGT2 inhibitors and SUs	7	RCTs	No serious risk of bias	No significant heterogeneity observed	No serious indirectness	No serious imprecision	Possible publication bias due to limited number of studies included	−0.28 (−0.35 to −0.20)	⊕⊕⊕O MODERATE
Difference of HbA1c change from 48∼52 weeks to 96∼104 weeks between SLGT2 inhibitors and SUs	7	RCTs	No serious risk of bias	No significant heterogeneity observed	No serious indirectness	No serious imprecision	Possible publication bias due to limited number of studies included	−0.11 (−0.19 to −0.04)	⊕⊕⊕O MODERATE
Difference of HbA1c change from 52 weeks to 208 weeks between SLGT2 inhibitors and SUs	2	RCTs	No serious risk of bias	No significant heterogeneity observed	No serious indirectness	No serious imprecision	Possible publication bias due to limited number of studies included	−0.22 (−0.34 to −0.10)	⊕⊕⊕O MODERATE
Difference of HbA1c change from 104 weeks to 208 weeks between SLGT2 inhibitors and SUs	2	RCTs	No serious risk of bias	No significant heterogeneity observed	No serious indirectness	No serious imprecision	Possible publication bias due to limited number of studies included	−0.12 (−0.25 to −0.01)	⊕⊕⊕O MODERATE

Abbreviations: GRADE: Grading of Recommendations, Assessment, Development and Evaluation; RCTs: Randomized controlled trials; MD: Mean difference; CI: Confidence interval; HbA1c: Hemoglobin A1c; SGLT2: Sodium-glucose co-transporter 2; SUs: Sulfonylureas. Specific reasons for each GRADE domain, including:Risk of bias: Downgraded if a significant proportion of studies had unclear or high risk of bias in key domains (e.g., random sequence generation, allocation concealment, or selective reporting).Inconsistency: Downgraded if substantial heterogeneity was observed (*I^2^* > 50%) and could not be explained by subgroup analyses or meta-regression.Indirectness: Evaluated but not downgraded, as all included studies directly assessed the population and outcomes of interest.Imprecision: Downgraded if confidence intervals were wide, overlapping no effect, or if the overall sample size was small.Publication bias: Assessed using funnel plots and Egger’s test; downgraded if significant asymmetry suggested potential bias.

### Comparing glycemic duration of SGLT2 inhibitors with Sus

**Figure 2.**
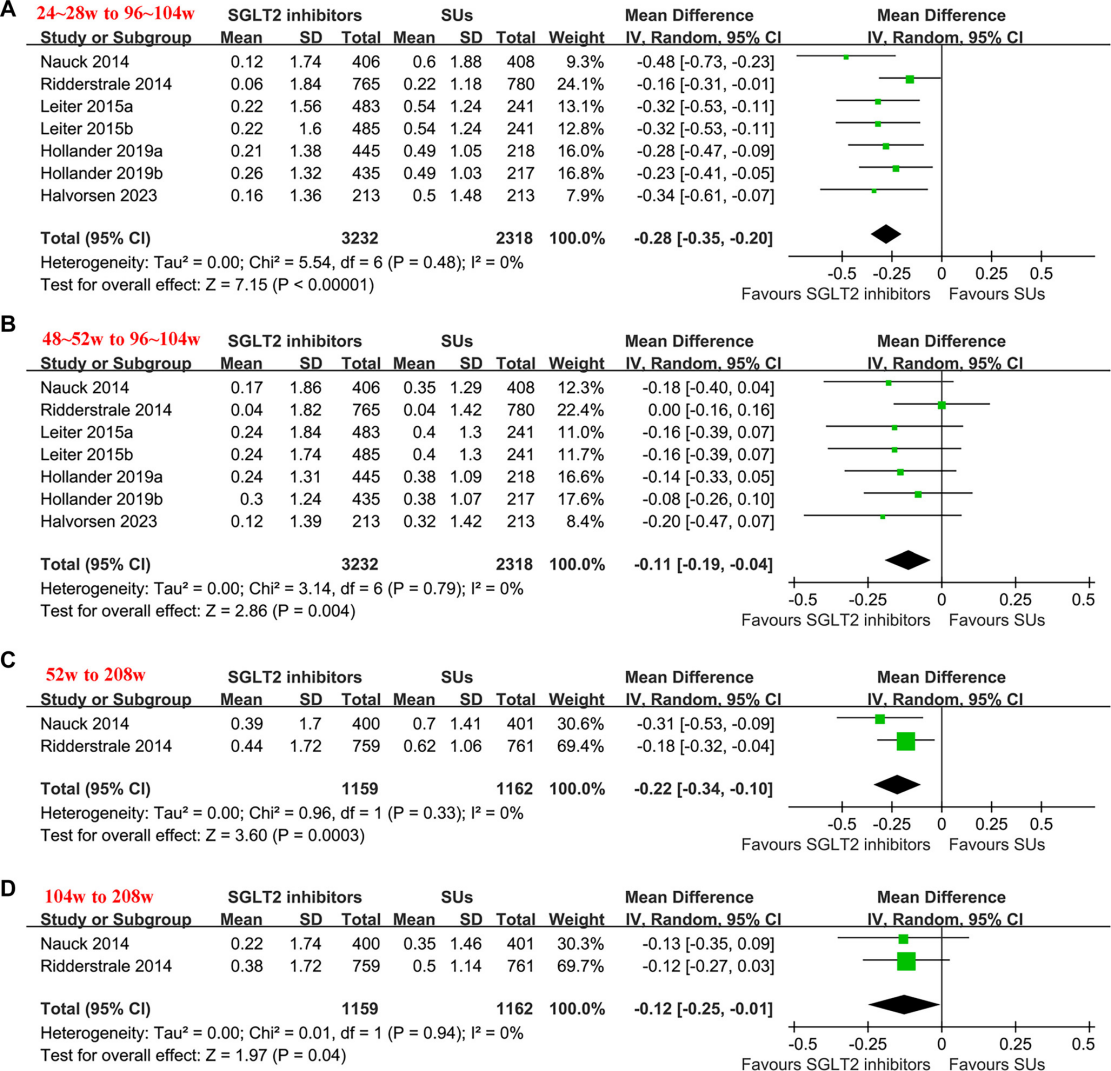
**Forest plots for the meta-analysis comparing the glycemic durability of SGLT2 inhibitors versus SUs in patients with T2DM**. (A) Change of HbA1c from 24–28 to 96–104 weeks in each group; (B) Change of HbA1c from 48–52 to 96–104 weeks in each group; (C) Change of HbA1c from 52 to 208 weeks in each group; (D) Change of HbA1c from 104 to 208 weeks in each group. Abbreviations: HbA1c: Glycated hemoglobin A1c; MD: Mean difference; CI: Confidence interval; SGLT2: Sodium-glucose co-transporter 2; SUs: Sulfonylureas; T2DM: Type 2 diabetes mellitus; IV: Inverse variance.

The pooled results of seven datasets from five RCTs [20, 21, 23, 25, 26] indicated that SGLT2 inhibitors were associated with a significantly smaller change in HbA1c from 24–28 weeks to 96–104 weeks (MD: −0.28%, 95% CI −0.35 to −0.20; *P* < 0.001; Figure 2A) and from 48–52 weeks to 96–104 weeks (MD: −0.11%, 95% CI −0.19 to −0.04; *P* ═ 0.004; Figure 2B), with no significant heterogeneity (both *I*^2^ ═ 0%). The summarized certainty of evidence, using the GRADE system, is presented in Table 3. We downgraded the evidence by one level due to the potential for publication bias stemming from the limited number of studies included. As a result, we judged the evidence to be of moderate certainty. Sensitivity analyses, conducted by excluding one dataset at a time, showed consistent results (MD for the change in HbA1c from 24–28 to 96–104 weeks: −0.31 to −0.26%, *p* all < 0.001; MD for the change in HbA1c from 48–52 to 96–104 weeks: −0.15 to −0.10%, *p* all < 0.05; Table 4). Further meta-analyses, which included two studies [22, 24], suggested that SGLT2 inhibitors also demonstrated better glycemic durability compared to SUs, as shown by the small changes in HbA1c from 52–208 weeks (MD: -0.22%; 95% CI −0.34 to −0.10; *P* < 0.001; I^2^ ═ 0%; Figure 2C) and from 104–208 weeks (MD: −0.12%; 95% CI −0.25 to −0.01; *P* ═ 0.04; I^2^ ═ 0%; Figure 2D). The certainty of evidence, summarized in Table 3, was also rated as moderate due to the potential for publication bias resulting from the limited number of studies included.

**Table 4:** Results of sensitivity analyses

**Dataset excluded**	**Changes of HbA1c from 24∼28 weeks to 96∼104 weeks**				**Changes of HbA1c from 48∼52 weeks to 96∼104 weeks**			
	**MD (95% CI)**	***P* values for effect**	***P* values for heterogeneity**	***I* ^2^**	**MD (95% CI)**	***P* values for effect**	***P* values for heterogeneity**	***I^2^***
Nauck, 2014	−0.26 [−0.34, −0.18]	<0.001	0.75	0%	−0.10 [−0.19, −0.02]	0.01	0.74	0%
Ridderstrale, 2014	−0.31 [−0.40, −0.23]	<0.001	0.75	0%	−0.15 [−0.23, −0.06]	0.001	0.98	0%
Leiter, 2015a	−0.27 [−0.36, −0.19]	<0.001	0.37	6%	−0.11 [−0.19, −0.02]	0.01	0.71	0%
Leiter, 2015b	−0.27 [−0.36, −0.19]	<0.001	0.37	7%	−0.11 [−0.19, −0.02]	0.01	0.71	0%
Hollander, 2019a	−0.28 [−0.37, −0.19]	<0.001	0.35	10%	−0.11 [−0.19, −0.02]	0.01	0.69	0%
Hollander, 2019b	−0.28 [−0.37, −0.19]	<0.001	0.39	5%	−0.12 [−0.20, −0.03]	0.006	0.70	0%
Halvorsen, 2023	−0.27 [−0.35, −0.19]	<0.001	0.38	6%	−0.10 [−0.19, −0.02]	0.01	0.75	0%

Abbreviations: MD: Mean difference; CI: Confidence interval; HbA1c: Hemoglobin A1c.

**Figure 3.**
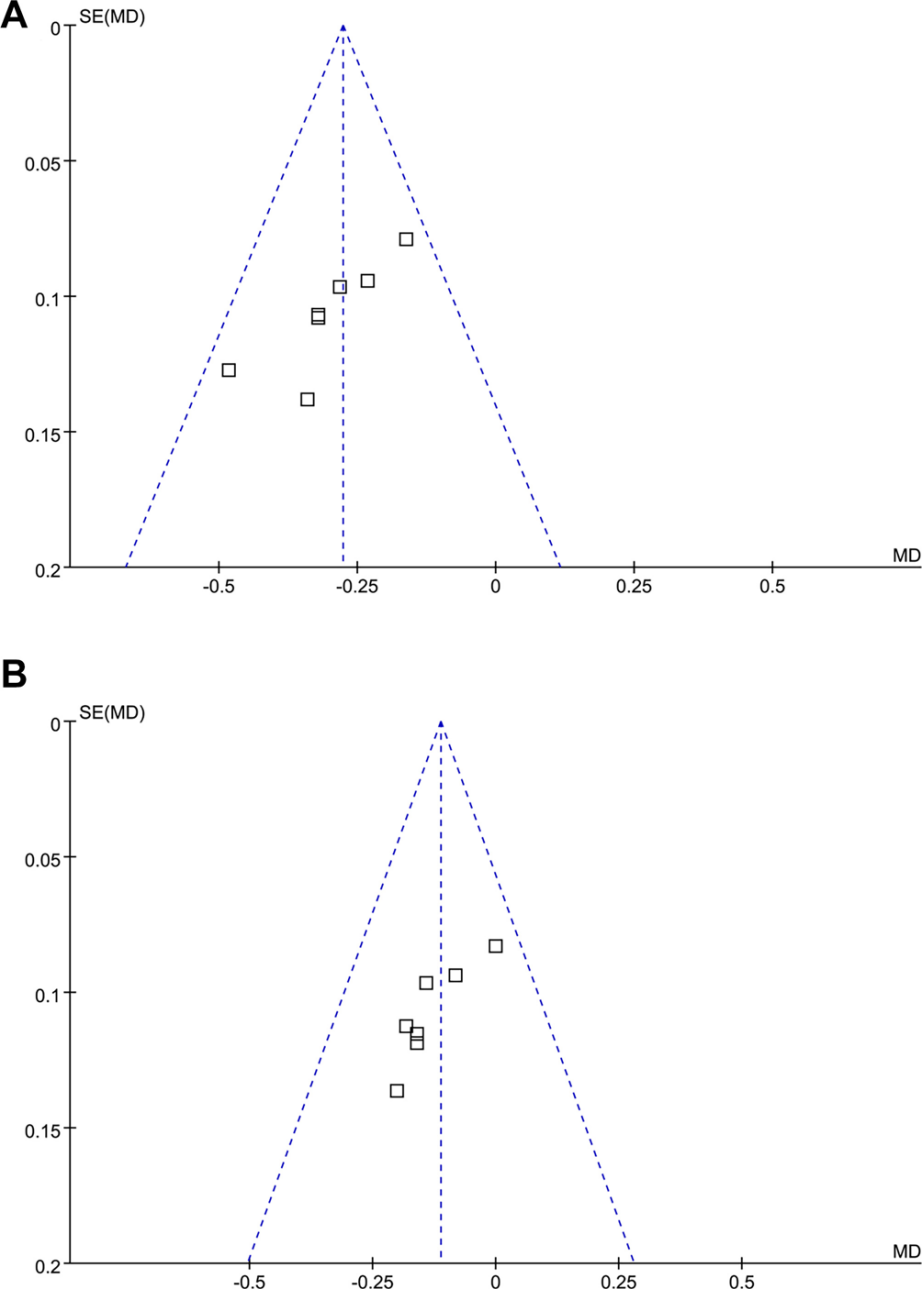
**Funnel plots evaluating the publication bias underlying the meta-analyses**. (A) Funnel plots for the meta-analysis of the change of HbA1c from 24–28 to 96–104 weeks in between SGLT2 inhibitors and SUs; (B) Funnel plots for the meta-analysis of the change of HbA1c from 48–52 to 96–104 weeks in between SGLT2 inhibitors and Sus. Abbreviations: HbA1c: Glycated hemoglobin A1c; SGLT2: Sodium-glucose co-transporter 2; SUs: Sulfonylureas.

### Publication bias

The funnel plots for the meta-analyses comparing the change in HbA1c from 24–28 weeks and 48–52 weeks to 96–104 weeks are shown in Figures 3A and 3B. These plots appear symmetrical upon visual inspection, suggesting a low risk of publication bias. Egger’s regression tests also indicated a low risk of publication bias (*P* ═ 0.47 and 0.51, respectively). However, the publication bias for the meta-analyses comparing the change in HbA1c from 52 and 104 weeks to 208 weeks could not be assessed, as only two studies were included for these outcomes. Furthermore, despite the symmetrical appearance of the funnel plots and the nonsignificant Egger’s tests (*P* ═ 0.47 and 0.51, respectively), the small number of trials included (*n* ═ 5) limits the statistical power to detect publication bias. Therefore, the presence of publication bias cannot be ruled out.

## Discussion

In this meta-analysis of head-to-head RCTs, we found that SGLT2 inhibitors demonstrated superior glycemic durability compared to SUs in patients with T2DM who were on background metformin therapy. Specifically, SGLT2 inhibitors were associated with significantly smaller increases in HbA1c from both early (24–28 weeks) and mid-term (48–52 weeks) follow-up to 96–104 weeks. Additionally, results from two RCTs with extended follow-up suggested that this benefit persisted through 208 weeks (4 years). These findings were consistent across sensitivity analyses and demonstrated low heterogeneity, indicating a robust and generalizable effect. Our study provides timely, comprehensive evidence on the long-term glycemic effectiveness of SGLT2 inhibitors relative to SUs, highlighting their value in the management of T2DM. The clinical relevance of these findings lies in the progressive nature of T2DM. While most therapies achieve good short-term glycemic control, the long-term sustainability of HbA1c reductions—referred to as glycemic durability—is a critical treatment goal [33]. Loss of glycemic control necessitates the escalation of therapy and increases the risk of diabetes-related complications [34]. In our analysis, the MD in HbA1c change from 24–28 to 96–104 weeks was −0.28% in favor of SGLT2 inhibitors. Though numerically modest, this difference represents a clinically meaningful benefit when sustained over time, especially considering the cumulative impact of hyperglycemia on vascular risk [35]. A similar trend was observed from 48–52 to 96–104 weeks (MD −0.11%) and in longer-term follow-ups (MD −0.22% from 52–208 weeks; MD −0.12% from 104–208 weeks), indicating that SGLT2 inhibitors not only maintain glycemic control better but may also delay disease progression more effectively than SUs. Several pharmacologic and molecular mechanisms may explain the superior durability of SGLT2 inhibitors. First, these agents reduce glucose through urinary excretion, a mechanism independent of β-cell function or insulin secretion [36]. This contrasts with SUs, which stimulate insulin release and impose chronic stress on β-cells, potentially accelerating their exhaustion and failure [37]. Second, SGLT2 inhibitors are known to reduce glucotoxicity by lowering both fasting and postprandial glucose levels, thereby alleviating β-cell stress and preserving endogenous insulin secretory capacity [38, 39]. Third, animal and human studies suggest that SGLT2 inhibitors may exert direct anti-inflammatory and antioxidant effects, improve mitochondrial function, and enhance β-cell survival. These mechanisms collectively contribute to better long-term glycemic control [18, 19]. In contrast, continued stimulation of insulin release by SUs, even in the presence of low glucose levels, may exacerbate β-cell apoptosis and reduce their long-term effectiveness [40]. The strengths of our study include a comprehensive and up-to-date literature search across four major databases and the inclusion of only high-quality, multinational, double-blind RCTs. All studies adhered to rigorous trial designs and were assessed as having low risk of bias across all domains. The meta-analysis incorporated seven datasets from five RCTs, encompassing over 5,500 participants, which strengthens the statistical power and external validity of the results. Moreover, we applied a clinically meaningful outcome—change in HbA1c from intermediate to final time points—which mirrors real-world patterns of treatment response [41]. The GRADE assessment rated the certainty of evidence as moderate for all outcomes, with downgrading only for potential publication bias due to the limited number of studies available. Nonetheless, several limitations should be acknowledged. First, the number of eligible RCTs was relatively small, and only two studies reported data beyond 104 weeks, limiting our ability to make definitive conclusions regarding long-term durability beyond four years. Second, while we pooled results across different agents within the SGLT2 inhibitor and SU classes, we could not determine whether specific drugs within each class performed better or worse than others. Future studies should explore potential class effects or agent-specific differences. Third, subgroup analyses based on patient comorbidities, such as cardiovascular disease, renal impairment, or obesity, were not feasible due to the lack of individual participant data. These factors could influence treatment response and should be investigated in future meta-analyses or patient-level pooled analyses. Lastly, although publication bias was not detected through funnel plot symmetry and Egger’s test for primary outcomes, the small number of included studies means this possibility cannot be fully excluded, as formal tests have limited power in such contexts. Given the superior glycemic durability of SGLT2 inhibitors, along with their well-established benefits on body weight, blood pressure, and cardiovascular and renal outcomes [16], they should be strongly considered as preferred second-line agents after metformin for patients with T2DM. While SUs remain a cost-effective option in many settings, their limited durability and risk of hypoglycemia must be carefully considered, particularly in younger patients with a long life expectancy or those at risk of hypoglycemia [42]. Our findings also support the inclusion of durability metrics in future clinical guidelines and cost-effectiveness analyses when evaluating antidiabetic therapies.

Looking forward, future research should address current knowledge gaps by conducting head-to-head trials comparing specific SGLT2 inhibitors and SUs across diverse patient subgroups and care settings. Moreover, real-world factors, such as medication adherence, treatment persistence, and the presence of comorbidities (e.g., cardiovascular or renal disease), may significantly impact the long-term glycemic durability of antidiabetic therapies. Observational studies suggest that SGLT2 inhibitors may lead to better long-term persistence and adherence compared to SUs, potentially enhancing their effectiveness beyond controlled trial settings [43, 44]. Incorporating real-world data, especially beyond the 4-year timeframe of existing RCTs, is essential for validating the durability benefits observed in clinical trials. Additionally, biomarker-driven studies exploring β-cell preservation and metabolic remodeling under SGLT2 inhibitor therapy could further our understanding of their disease-modifying potential.

## Conclusion

In conclusion, this meta-analysis provides strong evidence that SGLT2 inhibitors offer superior long-term glycemic durability compared to SUs in patients with type 2 diabetes who are on metformin therapy. These findings support the preferential use of SGLT2 inhibitors as second-line agents and contribute to the evolving paradigm of durable, pathophysiology-based treatment strategies in diabetes care.

## Supplemental data

**Supplemental File 1.** Detailed search strategy for each database

**PubMed (MEDLINE)**

((“sodium-glucose transporter 2 inhibitors”[MeSH Terms] OR “sodium glucose transporter 2 inhibitor” OR “sodium glucose transporter ii inhibitor” OR “SGLT 2 inhibitor” OR “SGLT-2 inhibitor” OR “SGLT2” OR “sodium glucose cotransporter 2 inhibitors” OR canagliflozin OR dapagliflozin OR empagliflozin OR ertugliflozin OR tofogliflozin OR bexagliflozin OR henagliflozin OR ipragliflozin OR licogliflozin OR luseogliflozin OR remogliflozin OR sergliflozin OR sotagliflozin) AND (“sulfonylurea compounds”[MeSH Terms] OR glimepiride OR glipizide OR gliclazide OR glibenclamide OR glyburide OR gliguidone OR sulfonylureas OR sulfonylureas) AND (“randomized controlled trial”[Publication Type] OR random* OR randomly OR randomized OR randomised))

**Embase (via Elsevier)**

(‘sodium glucose transporter 2 inhibitor’/exp OR ‘sodium glucose transporter ii inhibitor’ OR ‘sglt 2 inhibitor’ OR ‘sglt-2 inhibitor’ OR ‘sglt2’ OR ‘sodium glucose cotransporter 2 inhibitor’ OR canagliflozin OR dapagliflozin OR empagliflozin OR ertugliflozin OR tofogliflozin OR bexagliflozin OR henagliflozin OR ipragliflozin OR licogliflozin OR luseogliflozin OR remogliflozin OR sergliflozin OR sotagliflozin) AND (‘sulfonylurea derivative’/exp OR glimepiride OR glipizide OR gliclazide OR glibenclamide OR glyburide OR gliguidone OR sulfonylureas OR sulfonylureas) AND (‘randomized controlled trial’/exp OR random*:ab,ti OR randomly:ab,ti OR randomized:ab,ti OR randomised:ab,ti)

**Cochrane Library (CENTRAL)**

(“sodium glucose transporter 2 inhibitor” OR “sodium glucose transporter ii inhibitor” OR “SGLT 2 inhibitor” OR “SGLT-2 inhibitor” OR “SGLT2” OR “sodium glucose cotransporter 2 inhibitors” OR canagliflozin OR dapagliflozin OR empagliflozin OR ertugliflozin OR tofogliflozin OR bexagliflozin OR henagliflozin OR ipragliflozin OR licogliflozin OR luseogliflozin OR remogliflozin OR sergliflozin OR sotagliflozin) AND (glimepiride OR glipizide OR gliclazide OR glibenclamide OR glyburide OR gliguidone OR sulfonylureas OR sulfonylureas) AND (random* OR randomly OR randomized OR randomised)

**Web of Science Core Collection**

TS ═ ((“sodium glucose transporter 2 inhibitor” OR “sodium glucose transporter ii inhibitor” OR “SGLT 2 inhibitor” OR “SGLT-2 inhibitor” OR “SGLT2” OR “sodium glucose cotransporter 2 inhibitors” OR canagliflozin OR dapagliflozin OR empagliflozin OR ertugliflozin OR tofogliflozin OR bexagliflozin OR henagliflozin OR ipragliflozin OR licogliflozin OR luseogliflozin OR remogliflozin OR sergliflozin OR sotagliflozin) AND (glimepiride OR glipizide OR gliclazide OR glibenclamide OR glyburide OR gliguidone OR sulfonylureas OR sulfonylureas) AND (random* OR randomly OR randomized OR randomised))

## Data Availability

All data generated or analyzed during this study are included in this published article.
